# Using phenotypic distribution models to predict livestock performance

**DOI:** 10.1038/s41598-019-51910-6

**Published:** 2019-10-25

**Authors:** M. Lozano-Jaramillo, S. W. Alemu, T. Dessie, H. Komen, J. W. M. Bastiaansen

**Affiliations:** 1 Wageningen University & Research Animal Breeding and Genomics, PO Box 338, 6700 AH Wageningen, The Netherlands; 2 International Livestock Research Institute, P. O. Box 5689, Addis Ababa, Ethiopia

**Keywords:** Ecological modelling, Agroecology

## Abstract

Livestock production systems of the developing world use indigenous breeds that locally adapted to specific agro-ecologies. Introducing commercial breeds usually results in lower productivity than expected, as a result of unfavourable genotype by environment interaction. It is difficult to predict of how these commercial breeds will perform in different conditions encountered in e.g. sub-Saharan Africa. Here, we present a novel methodology to model performance, by using growth data from different chicken breeds that were tested in Ethiopia. The suitability of these commercial breeds was tested by predicting the response of body weight as a function of the environment across Ethiopia. Phenotype distribution models were built using machine learning algorithms to make predictions of weight in the local environmental conditions based on the productivity for the breed. Based on the predicted body weight, breeds were assigned as being most suitable in a given agro-ecology or region. We identified the most important environmental variables that explained the variation in body weight across agro-ecologies for each of the breeds. Our results highlight the importance of acknowledging the role of environment in predicting productivity in scavenging chicken production systems. The use of phenotype distribution models in livestock breeding is recommended to develop breeds that will better fit in their intended production environment.

## Introduction

In livestock production systems of the developing world, native breeds are known to be locally adapted to specific environmental conditions^1,2^. This adaptability allows these breeds to fulfil a key role in providing nutrition and income to the rural and peri-urban households, particularly under low input management systems. However, these local breeds exhibit low productivity when compared to exotic breeds^3^. As a means to improve local meat and egg production, the African continent has witnessed the introduction of exotic chicken breeds^4^. Nevertheless, after more than 40 years of exotic breed introduction most attempts have been unsuccessful mainly because of the non-adaptability of these breeds to the local environmental conditions, and the prevailing low input production systems^5–7^.

Nowadays, the poultry industry in most developing countries is divided in three systems, village, semi-intensive and intensive. Distinctions between these production systems are based on the breeds (indigenous or introduced), but also on the management conditions^8^. Generally, introduced commercial breeds are kept under better housing and feeding conditions in the semi-intensive and intensive systems, while smallholders keep their indigenous breeds under semi-free range, scavenging feeding conditions in the village systems^2,8,9^. Birds in the village systems rely on foraging for water, house wastes, insects, snails, earthworms, and other resources where the availability is highly dependent on the local environment and season^1,8^.

There are a few commercial breeds that have been specifically developed for production in (semi-) tropical conditions. Examples include the Kuroiler, a hybrid chicken from India^10^, Sasso, a breed originating from France^11^, and the Koekoek, a breed developed in South Africa^12^. These breeds are developed to be successful and more productive in low maintenance, tropical systems. However, there is a lack of understanding of how these tropically adapted commercial breeds will perform across the wide range of environmental conditions encountered in sub-Saharan Africa and under diverse smallholder systems.

In a recent study, we used predictive habitat distribution models on the Koekoek breed, to predict areas of potential suitability for this breed in Ethiopia. We also identified which environmental conditions explain the breeds’ distribution in the country^13^. For that study no information on the productivity of the breed was taken into account, as only information about the location of the breed was available.

An approach to take the productivity into account, is the use of phenotype distribution models. These models were recently introduced in ecology studies to capture the response of phenotypic traits as a function of environmental conditions^14^. As the environment plays an important role in scavenging systems, this model can also have value to predict the productivity of a breed in a specific region. The application of phenotypic distribution models to understand the role of the environment in livestock productivity is novel and has not yet been implemented.

Due to the influence of environmental factors on animal performance, research on the mechanisms of livestock adaptation to challenging environments is urgently needed^15^. The African Chicken Genetic Gains project (ACGG; https://africacgg.net/) is testing the performance of different chicken breeds in smallholders’ households. ACGG is an Africa-wide collaboration research program led by the International Livestock Research Institute (ILRI) and Bill and Melinda Gates Foundation funded, that aims to improve chicken productivity to benefit rural smallholder households. Knowledge on how the productivity of these introduced chicken breeds is affected by the environment may allow predictions on how they would respond to specific agro-ecologies, and to tailor breeding programs to collect performance data in the most informative environments.

Our main objective was to predict the response of productivity traits of different introduced breeds, as a function of the environment where they were introduced. Using performance data on five chicken breeds from the ACGG project in Ethiopia, the association with environmental parameters was established using phenotypic distribution models. Predictions for productivity traits were made to establish suitable areas for breed distribution. Such predictions could provide insightful information for breeding companies or farmers to decide which areas can be suitable to introduce a breed, or which breed will be better suited for a specific environment.

## Materials and Methods

Live body weight data from the five breeds tested in Ethiopia was obtained from the ILRI datasets portal. For details on ACGG see https://africacgg.net/. Environmental variables that characterize the different agro-ecological zones in Ethiopia were obtained from WorldClim^16^ and the Harmonized World Soil Database v 1.2^17^. All data cleaning and analyses were undertaken in R version 3.5.1^18^ running on RStudio version 1.1.383^19^. The experimental protocols and ethical guidelines were approved by the ILRI Institutional Research Ethics Committee (ILRI IREC) under reference number ILRI-IREC2015-08. ILRI IREC is accredited by the National Commission for Science, Technology and Innovation (NACOSTI) in Kenya. All the methods were performed in accordance with the relevant guidelines and regulations.

### Chicken breeds

The ACGG program identified four exotic chicken breeds presumably tolerant to sub-Saharan African environmental conditions (Koekoek, Kuroiler, Sasso, and Sasso-RIR), and in 2016 fertile eggs were imported into Ethiopia. A locally improved breed named Horro was also used in parallel to the exotic breeds. The latter one assumed to be locally adapted, and genetically improved for growth and egg production in the environmental conditions of the low input systems characterizing the smallholders farms in Ethiopia^20^.

**Figure 1:**
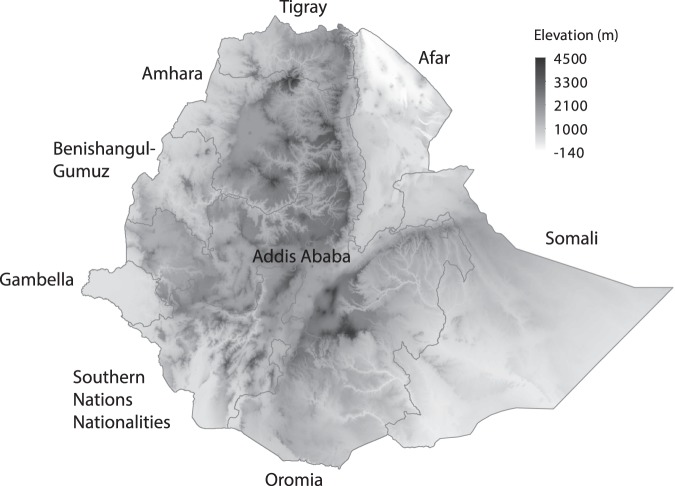
Map of Ethiopia showing its regional states.

The five breeds were tested in 63 villages, divided over five regions across Ethiopia (Addis Ababa, Amhara, Oromia, Southern Nations Nationalities, and Tigray; see Fig. 1). A total of 1393 households received approximately 25 six-week old chicks per household. All breeds were tested in all the villages, but only one breed was tested in each household. The introduced chickens were exposed to the same environmental and management conditions as the local breeds already kept by the farmers. The breed distribution started in August 2016, and data collection ended in January 2018.

### Breed and phenotypic data

Households were georeferenced, and each breed was randomly assigned to a household. Chickens were delivered at 6 weeks of age. Traditionally in Ethiopia, farmers sell the male chickens when they reach 2 kg, which is around 20 weeks. For male chickens, weight data was collected until 20 weeks of age. Females are kept longer for egg production, therefore, weight data for females was collected until 72 weeks of age.

### Data cleaning

In each household, body weight data was collected every two weeks as a group measure. Taking into account the number of birds weighted, we converted the group weight to average individual body weight. After calculating the average individual weight, we first eliminated all observations with weight less than 50 grams. We deleted the households with more than 30 birds reported per household. We also deleted those observations where age was less than 6 weeks. For males we used male body weight during the growing phase (weeks 14–19) and for females we divided the data into growing phase (weeks 14–19), and adult phase (weeks 20–72). Not all the weeks had data for all of the households. Therefore, we standardized the body weight in each household to the average value of week in each age phase for all breeds for males and females independently. The average week value for the growing phase for males and females was 16.54 and 16.52 respectively. For females during the adult phase this was 41.81. Then, the following linear model was fitted:

1 $${\boldsymbol{y}}=H+{\bf{W}}+{\boldsymbol{e}}$$

where ${\boldsymbol{y}}$ is the vector with the average bird weights, **H** is the household at the week of measurement, **W** is the week of measurement, and $\,e$ is the vector containing the random residual error. We assumed that within breeds all chickens had the same body weight at the time of hatching and fitted a model with common intercept and different slope per household. For each household for each age phase independently, we calculated the least squares means (LSmeans), using the emmeans package in R^21^.

### Environmental data

The introduced chickens were kept in low input systems along with indigenous chicken. Some feed supplementation was given by farmers at their discretion. The supplemented feed was either grown by the farmers or bought externally, the rest of the feed intake was from scavenging. The introduced chickens had to cope with the same environmental conditions as the indigenous chickens, where food availability depends on seasonality. Therefore, we used a total of 21 variables at a 1 km by 1 km resolution (Table 1). The environmental data, representing current conditions included 19 bioclimatic variables and an elevation variable. These 20 variables represent trends in temperature, seasonality and precipitation, variables that are expected to have an influence on the chicken productivity and food availability. Smallholders in Ethiopia tend to have crops in their households, which has an influence on what is fed to their livestock. To represent this link between human intervention and agriculture^1,4^ we used an additional layer, the total cultivated land. This variable represents the percentage of area that is used in agriculture, therefore having an impact on the food availability.

**Table 1:** Environmental variables used to build the models.

Variables	Source
Annual Mean Temperature	WorldClim
Mean Diurnal Range (Mean of monthly (max temp - min temp))	WorldClim
Isothermality (BIO2/BIO7) (* 100)	WorldClim
Temperature Seasonality (standard deviation *100)	WorldClim
Max Temperature of Warmest Month	WorldClim
Min Temperature of Coldest Month	WorldClim
Temperature Annual Range (BIO5-BIO6)	WorldClim
Mean Temperature of Wettest Quarter	WorldClim
Mean Temperature of Driest Quarter	WorldClim
Mean Temperature of Warmest Quarter	WorldClim
Mean Temperature of Coldest Quarter	WorldClim
Annual Precipitation	WorldClim
Precipitation of Wettest Month	WorldClim
Precipitation of Driest Month	WorldClim
Precipitation Seasonality (Coefficient of Variation)	WorldClim
Precipitation of Wettest Quarter	WorldClim
Precipitation of Driest Quarter	WorldClim
Precipitation of Warmest Quarter	WorldClim
Precipitation of Coldest Quarter	WorldClim
Elevation	WorldClim
Total cultivated land	Harmonized World Soil

### Phenotypic variation models

In ecology, regression analyses are often used to explain the relationship between ecological data and to build prediction models. Ecological data can sometimes be complex showing non-linear relationships and/or spatial and temporal correlation. To address these problems, more flexible, but still complex models are often used, such as generalized additive models (GAMs). GAMs can identify nonlinear approaches, and still generate easy to interpret relationships. However, including too many covariates will lead to overfitting, which decreases the prediction accuracy. Therefore, several machine learning algorithms, such as boosting, have been used to increase the prediction accuracy of standard regression models^22^.

To model the relationship between environmental variables and chicken phenotypes, the values of the 21 environmental variables for each of the georeferenced household locations were used as predictor variables. The response variable was the LSmeans for body weight per household for each of the breeds at the average week for each growing phase. To increase prediction accuracy, gradient boosting, a machine learning algorithm, was applied to a GAM. Gradient boosting is an iterative process, where models are built in a step-wise fashion, starting from weak prediction models that fit the data poorly (base-learners), and by learning from the previous step, the fitting is improved in the next iteration^22^. All the models were built using all the environmental variables as base-learners. Boosting algorithms use as tuning parameter a stopping iteration, which determines the optimal point of where the algorithm should stop before convergence (e.i. early stopping). This choice of stopping iteration becomes crucial, as it prevents overfitting the data and improves the accuracy. The model for each breed was run independently, and a separate stopping iteration was determined for each model using the method described in Mayr, *et al*.^23^. The predicted body weight was represented in a heatmap that covers all the regions of the country were chickens are known to be kept. Desert areas (Somali and Afar regions (see Fig. 1) were excluded from the predictions, as the environmental conditions in these regions lay outside the range of the environmental conditions in which the chickens where tested (Supplementary Tables S1 and S2).

The variable of highest contribution in building each of the models was obtained. For this, the in-bag risk reductions per boosting step of a fitted model are accumulated individually for each variable (base-learner) contained in the model. This quantifies the individual variable contribution to risk reduction of each variable (base-learner), and can be used to compare the importance of different variable in the model^24^. To quantify the precision of our predictions, we estimated the correlation between the predicted value obtained from the phenotypic distribution models, and the LSmeans per household in each region. The LSmeans are the observed values for each of the households. With the model we predict the performance. If the predictions are precise, then the correlation between the predictions and the LSmeans would be high. Data cleaning, modelling the phenotypic variation and importance variable selection were done using the package mboost in R^24^.

## Results

### Estimated body weights

LSmeans for male body weights were estimated for week 16.54 (growing phase; Table 2). The heaviest breed was the Sasso with a mean estimated body weight of 1035.7 g, and the lightest was the Horro with a mean of 635.3 g. LSmeans for female body weights were estimated for week 16.52 (Table 3). The heaviest breed was the Koekoek with a mean estimated body weight of 1280.2 g, and the lightest was the Horro with a mean estimated weight of 738.6 g. LSmeans for female body weights in week 41.81 (adult phase) were highest for the Sasso-RIR with a mean weight of 2863.3 g, and the lightest remained to be the Horro with a mean estimated body weight of 2250.8 g (Table 4).

**Table 2:** Minimum, maximum and mean estimated (LSmeans) male body weight for each of the five breeds across Ethiopia during the growing phase (week 16.54).

Breed	Minimum estimated body weight (g)	Maximum estimated body weight (g)	Mean estimated body weight (g) (S.E)
Horro	403.9	1119.2	635.3 (12.3)
Koekoek	401.9	1675.4	842.5 (11.2)
Kuroiler	436.8	1678.3	919.9 (16.3)
Sasso	504.3	2173.2	1035.7 (21.2)
Sasso-RIR	398.4	1554.8	745.6 (11.6)

**Table 3:** Minimum, maximum and mean estimated (LSmeans) female body weight for each of the five breeds across Ethiopia during the growing phase (week 16.52).

Breed	Minimum estimated body weight (g)	Maximum estimated body weight (g)	Mean estimated body weight (g) (S.E)
Horro	515.6	1058.1	738.6 (15.9)
Koekoek	534.4	1658.6	1280.2 (10.5)
Kuroiler	565.5	1227.3	896.3 (17.3)
Sasso	569.9	1415.2	1081.2 (12.7)
Sasso-RIR	512.4	1500.9	1041.1 (11.3)

**Table 4:** Minimum, maximum and mean estimated (LSmeans) female body weight for each of the five breeds across Ethiopia during the adult phase (week 41.81).

Breed	Minimum estimated body weight (g)	Maximum estimated body weight (g)	Mean estimated body weight (g) (S.E)
Horro	1323.1	2910.8	2250.8 (8.8)
Koekoek	1961.4	3149.8	2703.9 (10.7)
Kuroiler	1565.5	3281.3	2796.1 (9.2)
Sasso	1726.0	3307.8	2821.2 (10.1)
Sasso-RIR	1995.4	3399.6	2863.3 (7.6)

### Predicted body weights

**Figure 2:**
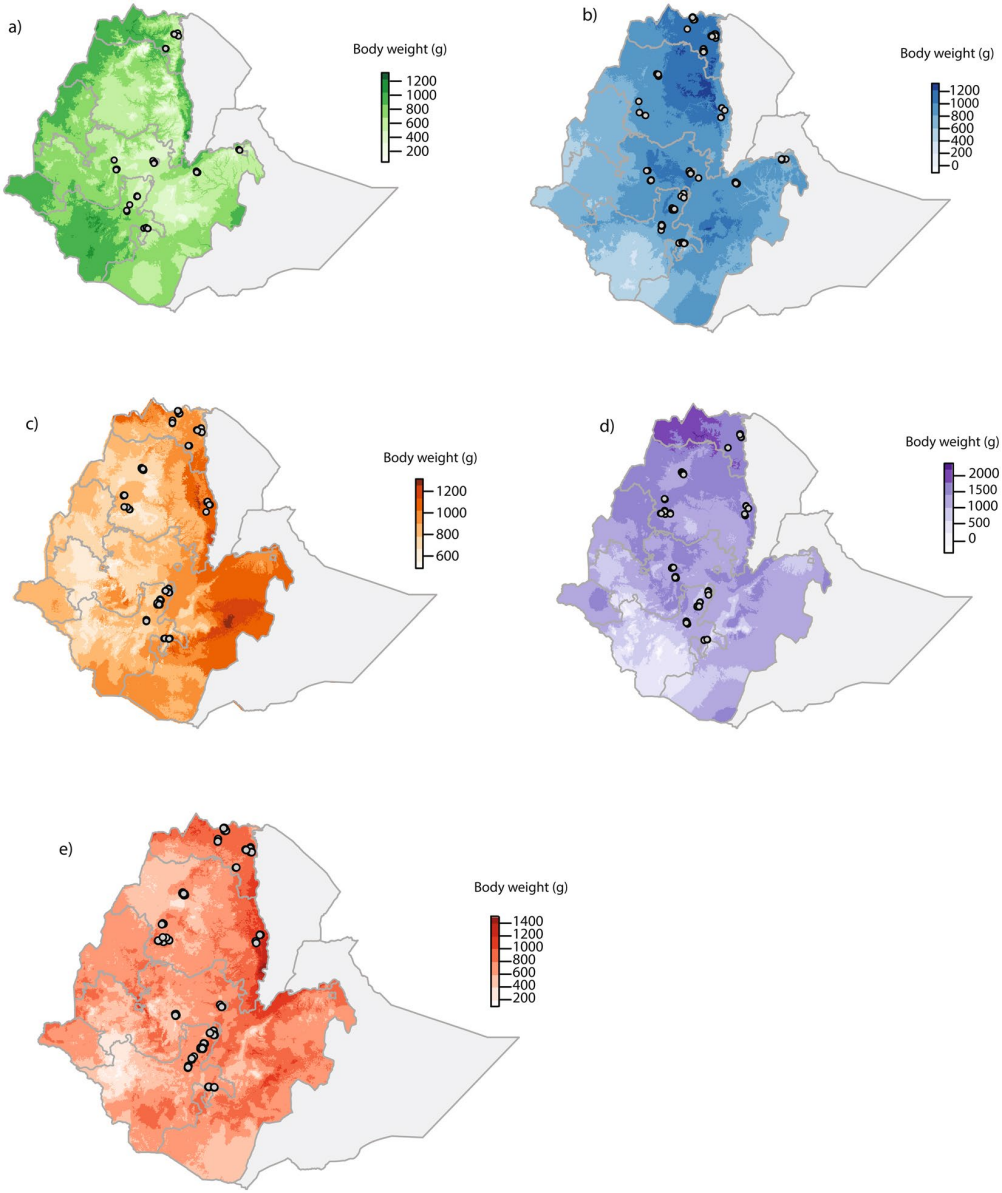
Predicted current phenotypic measures for male body weight in grams during the growing period (weeks 14 to 19) for (**a**) Horro, (**b**) Koekoek, (**c**) Kuroiler, (**d**) Sasso, and (**e**) Sasso-RIR. Circles in the maps denote the household locations where the phenotypic data was collected. Color scales in each of the maps reflect the predicted body weight, where darker colors indicate higher live weight predicted.

**Figure 3:**
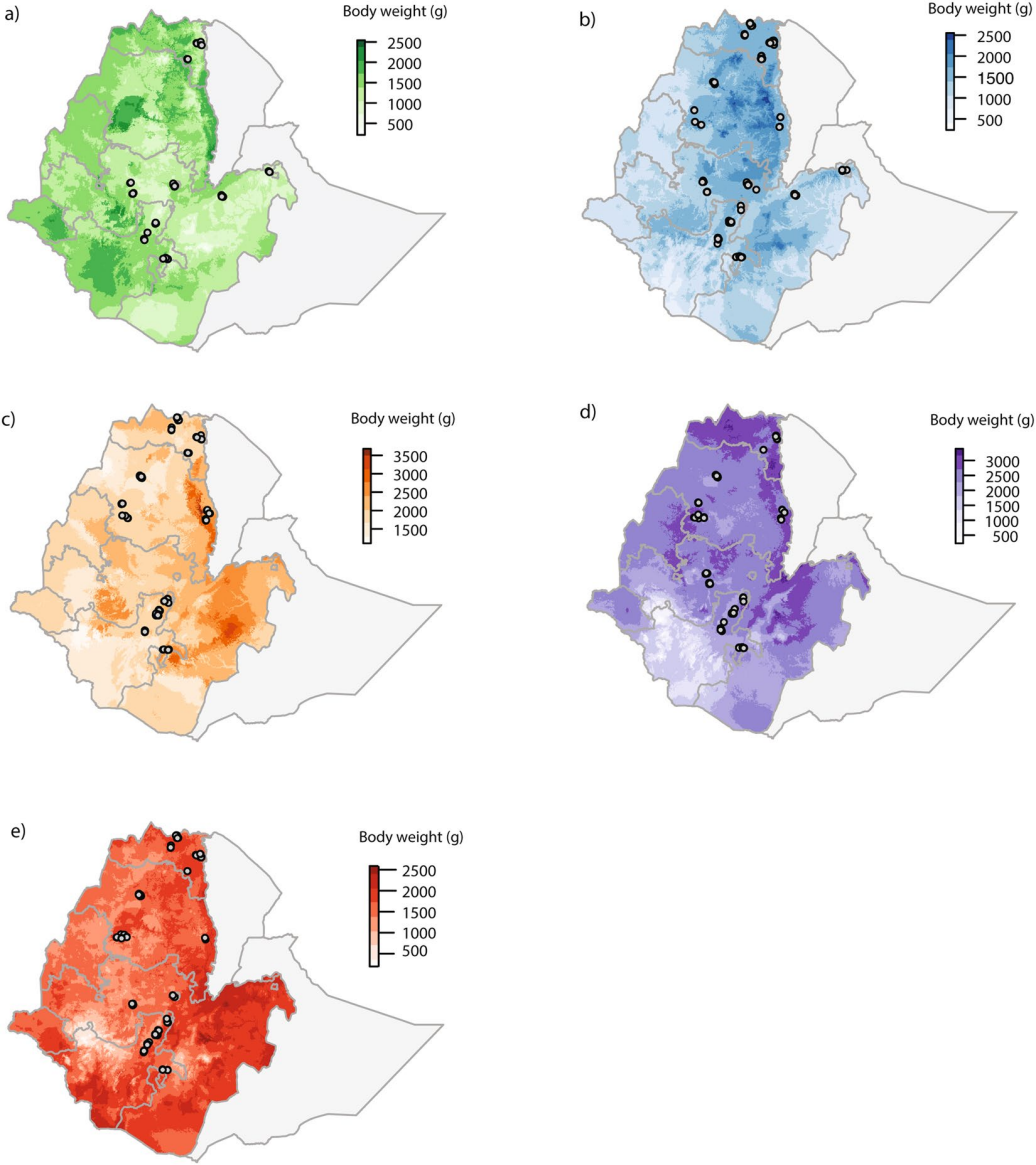
Predicted current phenotypic measures for female body weight in grams during the growing period (weeks 14 to 19) for (**a**) Horro, (**b**) Koekoek, (**c**) Kuroiler, (**d**) Sasso, and (**e**) Sasso-RIR. Circles in the maps denote the household locations where the phenotypic data was collected. Color scales in each of the maps reflect the predicted body weight, where darker colors indicate higher live weight predicted.

**Figure 4:**
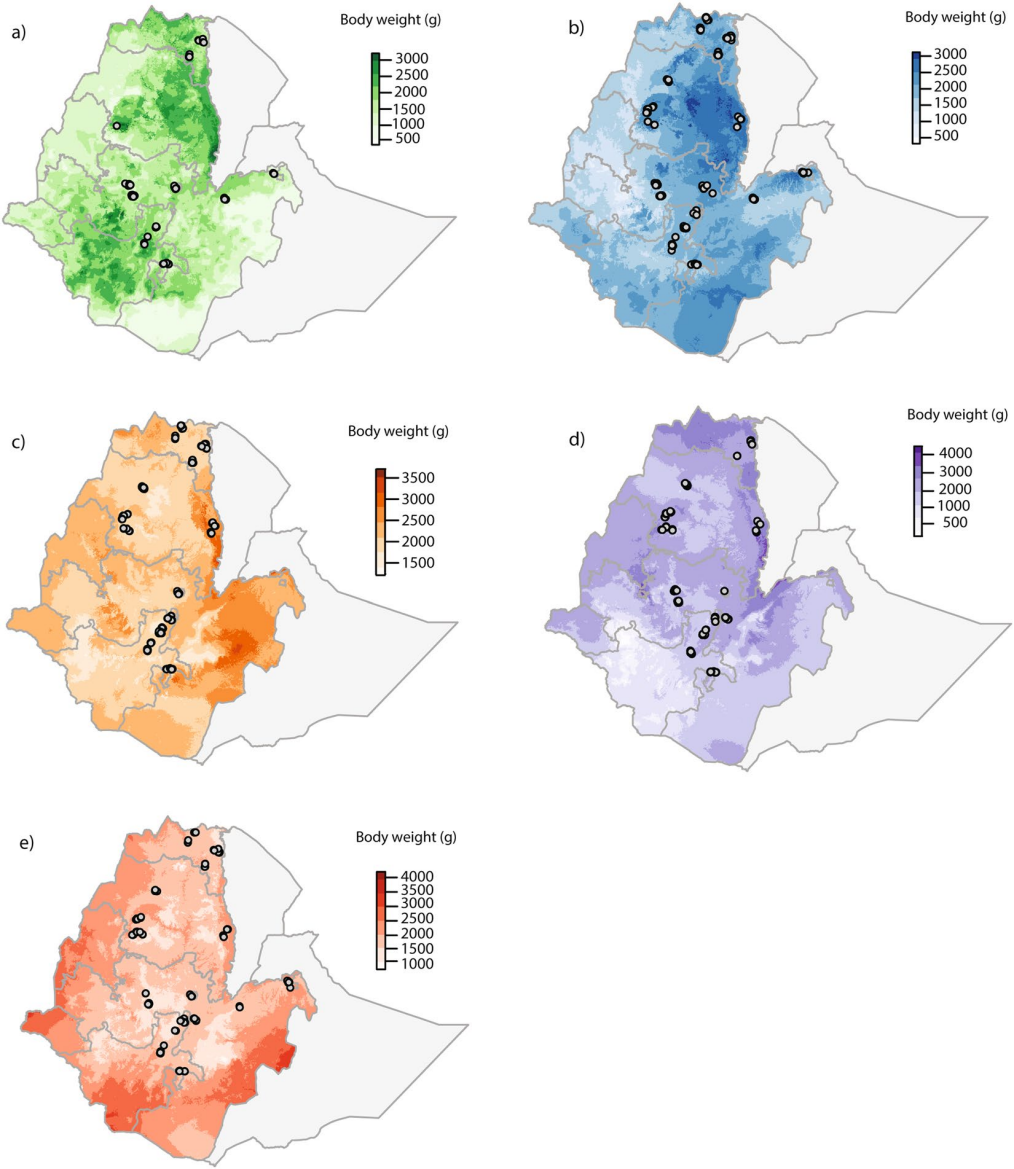
Predicted current phenotypic measures for female body weight in grams during the adult period (weeks 20 to 72) for (**a**) Horro, (**b**) Koekoek, (**c**) Kuroiler, (**d**) Sasso, and (**e**) Sasso-RIR. Circles in the maps denote the household locations where the phenotypic data was collected. Color scales in each of the maps reflect the predicted body weight, where darker colors indicate higher live weight predicted.

Predicted body weights varied considerably between breeds across Ethiopia. In general, for the growing phase of males and females, the models predicted for the Sasso breed to have the highest body weight with 2185.2 g for males and 3862.8 g for females (Fig. 2; Tables 2 and 5). The Horro breed is predicted to have the lowest body weight with 1327 g for males and 2540.3 g for females (Fig. 3; Tables 5 and 6). The female body weight predicted for the adult phase was the highest for the Sasso breed with 4330.8 g. The lightest breed predicted was the Horro with 3074.6 g (Fig. 4; Table 7).

**Table 5:** Minimum and maximum male body weights predicted for growing phase. The most important environmental variable for building the model and region predicted for higher body weights is shown per breed.

Breed	Minimum predicted weight (g)	Maximum predicted weight (g)	Environmental variable of importance	Region with higher body weights predicted
Horro	216.8	1327	Maximum temperature of warmest month	Tigray
Koekoek	161.1	1381.3	Mean diurnal range	Amhara
Kuroiler	656.4	1448.8	Precipitation of coldest quarter	Oromia
Sasso	282	2185.2	Mean diurnal range	Tigray
Sasso-RIR	270.5	1558.6	Isothermality	Amhara

**Table 6:** Minimum and maximum female body weights predicted for growing phase. The most important environmental variable for building the model and region predicted for higher body weights is shown per breed.

Breed	Minimum predicted weight (g)	Maximum predicted weight (g)	Environmental variable of importance	Region with higher body weights predicted
Horro	292.3	2540.3	Temperature annual range	Tigray
Koekoek	186.1	2635.5	Precipitation of wettest month	Amhara
Kuroiler	1022	3545.1	Precipitation of wettest month	Oromia
Sasso	524.6	3862.8	Annual temperature range	Tigray
Sasso-RIR	341.1	2556.8	Precipitation of driest month	Oromia

**Table 7:** Minimum and maximum female body weights predicted for adult phase. The most important environmental variable for building the model and region predicted for higher body weights is shown per breed.

Breed	Minimum predicted weight (g)	Maximum predicted weight (g)	Environmental variable of importance	Region with higher body weights predicted
Horro	388.1	3074.6	Precipitation of driest month	Amhara
Koekoek	225.1	3344.5	Precipitation of warmest quarter	Amhara
Kuroiler	1107.5	3746.2	Precipitation of warmest quarter	Oromia
Sasso	635.7	4330.8	Temperature annual range	Tigray
Sasso-RIR	730	4154.6	Elevation	Gambella

To assess the precision of our predictions, correlations between the predicted values from the phenotypic variation models and the household LSmeans were estimated for each growing phase and breed within each of the regions. There is considerable variation within region for all breeds and growing phases, both in estimated and predicted weights (Supplementary Figs S1–S3). The correlation estimates have a median value of 0.61 but vary by breed and region ranging from 0.18 to 0.89. As suggested by Bonett and Wright^25^, to better estimate a correlation, the sample size should be greater than 25. Therefore, the correlations were only estimated for those regions where the total number of households for each breed was greater than 25. For the lowest number of households, N = 28, the S.Es were 0.098 and 0.103. For the highest number of household, N = 155, the S.E was 0.016. (Supplementary Table S3; Supplementary Figs S1–S3).

### Region suitability

Predictions of optimal region per breed, and the most important variable in building the model are given in Tables 5, 6 and 7. Results for male and female body weights during the growth phase and adult weights were very similar. Sasso always had the highest predicted body weights in Tigray. Koekoek had the highest weights in Amhara, while Kuroiler had the highest weights in Oromia. For Horro and Sasso-RIR, the pattern was less clear. Horro was predicted to perform best in either Amhara or Tigray for males and females. For Sasso-RIR there was no clear single region where the breed was expected to perform best for all sex and age groups.

### Variables contributing to the models

The individual contribution from each environmental variable to build the best model were identified. The environmental variables of strongest influence for the predicted male body weight distribution were different for each breed. Patterns of male body weights for Horro, Koekoek, Sasso and Sasso-RIR were best predicted by variables associated with temperature. For Kuroiler, precipitation of the coldest quarter was the variable that had the strongest influence on the distribution (Table 5). For female body weights during the growing phase, the patterns of the body weight distribution for Horro and Sasso were again best predicted by temperature related variables. However, for Koekoek, Kuroiler and Sasso-RIR, precipitation variables had more influence on the distribution (Table 6). For female body weights during the adult phase, the body weights distribution patterns for Horro, Koekoek and Kuroiler were best predicted by variables associated with precipitation. For Sasso and Sasso-RIR, temperature annual range and elevation respectively had the largest influence on the body weights distribution (Table 7).

## Discussion

We used phenotypic distribution models, implementing gradient boosting, a machine learning technique, to increase the predictive ability of GAMs. Machine learning procedures improve the accuracy of the models, by addressing overfitting, and variable selection simultaneously. Broadly, our results demonstrate the importance of understanding how different breeds respond to environmental conditions, and how different environmental variables can influence their productivity.

In Ethiopia, the Horro breed was characterized and genetically improved for growth traits^6,20^. The breeding program was conducted under an intensive management system, including high quality poultry houses, formulated feed, and vaccination. The ACGG program is the first attempt to test the ability of this breed to adapt to on-farm environment and management conditions^20^. Even though our results show higher body weights than previously reported for the breed^26^, compared to the introduced commercial lines they exhibit lower growth performance. The Horro breed originates in the Oromia region, at the Horro district at altitudes ranging between 2580 to 2810 meters above sea level (masl) in cool wet highlands. The average annual temperature is 13.3 °C, and the seasons can be divided in three; a main rainy season, a dry season, and a short rainy season. The seasonal variety in the Horro district can explain why the Horro breed seems to perform better in low humidity areas with low levels of precipitation. A recent study showed that during wet periods, the Horro chickens are prone to die due to disease, and survive better in dry seasons^27^.

The Sasso breed originates from the south of France in warm, and dry areas. In accordance to what we find here, we see that the Sassos’ performance across ages is linked to temperature. Different studies have shown, in line with our findings, that the Sasso breed was more productive than the indigenous breeds in Ethiopia^28^. We identified that temperature-associated variables have an influence on the Sasso predicted body weight distribution during all the age phases evaluated. The Sasso-RIR tested in Ethiopia was obtained through a private poultry farm, were crosses were made to generate the breed for the ACGG program^28^. Therefore, no other studies have been reported using the Sasso-RIR cross to evaluate performance under scavenging conditions. For Sasso-RIR, different environmental variables are responsible for shaping the predicted body weight distribution depending on the age phase, suggesting that the breeds response to the agro-ecology depends on age. In comparison, the pure breed Sasso, was predicted to be heavier than the crossbred Sasso-RIR for the male and female growing periods. For the adult females, the prediction was similar for both breeds. It should be noted that the body weight patterns and the variables of importance in building the models are different between these two breeds, suggesting that both breeds may have been adapted to different environmental conditions. As this is the first report of the Sasso-RIR cross in on-farm testing, we encourage the continuation of testing Sasso and Sasso-RIR in a broader scale. Our results show that these breeds outperform the others, suggesting that they both cope with low-input conditions.

The Kuroiler is a dual-purpose breed developed in India under humid conditions to perform in low maintenance systems. Studies have shown its capacity to adapt to tropical countries and significantly outperform the local breeds on scavenging conditions^10^. In this study, this breed showed higher estimated body weights compared to the locally improved Horro. For the phenotypic prediction models, for all of the age phases, the body weight distribution was influenced by environmental variables linked to precipitation. Rainy season has been reported preferred for farmers to rear chickens, as high precipitation increases the vegetation and lowers predation^27^. It also shows that the humid origin of the breed plays a role in the productivity of the breed.

The Koekoek breed is of South African origin, descending from a cross between three different breeds. This breed has been of popular use among rural farmers in African countries for egg and meat production. In a previous study using predictive habitat distribution models we showed that the Amhara region, and areas with colder temperatures and bigger fluctuations in annual mean temperature were the most suitable areas for the survival of the Koekoek breed^13^. In accordance, the Amhara region and areas with higher temperature fluctuations are now predicted to have higher body weights for Koekoek males and females. Here we show, in contrast to previous results, that precipitation during the wettest and warmer periods have an influence on the predicted distribution of the breed for body weight. Even though both predictive models show temperature fluctuation to be an important variable, precipitation seems to also play a role in shaping the variation of the phenotype. Dissimilarities in the important variables that shape the predicted distribution of the breed based on presence versus the phenotype, suggests that different processes can determine the range of the Koekoeks’ breed performance distribution. We believe that the use of quantitative data in phenotypic distribution models gives a more accurate result compared to the presence only models (distribution models), as the former takes into account more detailed information about how the environment influences the life history traits of the breed.

Here we show that there is variation in the body weights estimated and predicted for each of the breeds for all the growth phases. In other words, that the breeds respond differently when exposed to the same environments. Correlations between predicted and estimated body weights are positive, but for some breeds and regions they are higher than others. These correlations were calculated using the locations where both a prediction and an LSmeans estimate were available. Therefore, the correlation of predictions to performance outside the locations of the households in the current dataset may be lower. For some breeds the correlations are similar between regions, however for others the values greatly differ, highlighting the variation in response within breeds to different agro-ecologies.

Variation in productivity among breeds can be attributed to the breeds’ origin, which can have an effect on the breeds intrinsic response to different environmental conditions. A recent study characterized the genetic diversity and identified genomic regions that presented adaptive advantages of chickens in Uganda and Rwanda^29^. Here the authors showed that the Kuroiler breed has a gene which does not occur in different African ecotypes. They indicate that this gene is associated with homeostatic regulatory functions such as response to hypoxia, cold, and starvation. They suggest that the presence of this gene may be representing the result of the selective pressures for stress tolerance during their development in India^29^.

Our results highlight the importance of taking environmental variables into account for different applications. Genotype by environment interaction (G x E), defined as the change in phenotypic performance of genotypes, relative to one another, when measured in different environments^30,31^ can be acknowledged using phenotypic distribution models. How sensitive a breed is to a particular environment has been of interest for many years in animal breeding. Accounting for which environmental variables have an influence on breed performance can help in predicting the occurrence of G x E interaction, and by establishing which areas within a country can be suitable for breed introduction. This knowledge can help breeding companies when choosing in which ecology they should introduce a breed, or it may help a farmer decide which breed to use in its own agro-ecology. Integrating habitat distribution models and phenotypic distribution models into animal breeding can provide a richer and more specific understanding of the environment’s role in explaining the variation in productivity of a breed in different circumstances.

## Conclusions

Agro-ecological diversity can be a challenge when developing breeding programs, particularly in tropical countries^27^. The use of gradient boosting GAMs is novel approach that can be applied to this problem. They can be used either by breeding companies or by farmers to include environmental information when deciding where to introduce a breed, or which breed is better suited for which environment. We encourage the use of these models in livestock studies to capture the breeds’ phenotypic variation as a response to different agro-ecological conditions. The approaches outlined in this paper can also be applied to different livestock breeds and locations, and could also be used to predict impacts of different climate change scenarios on change of productivity.

## Supplementary information

Supplementary material

## Data Availability

The datasets generated during and/or analysed during the current study are available from the corresponding author on reasonable request.
