# Supplementary material for: Using phenotypic distribution models to predict livestock performance
Source: Sci Rep. 2019 Oct 25;9:15371. doi: 10.1038/s41598-019-51910-6 (PMC6814727; doi:10.1038/s41598-019-51910-6)

**Using phenotypic distribution models to predict livestock performance**

M. Lozano-Jaramillo*, S.W. Alemu, T. Dessie, H. Komen, J. W. M. Bastiaansen

*Corresponding author, e-mail: [maria.lozanojaramillo@wur.nl](mailto:maria.lozanojaramillo@wur.nl)

**Table S1.** Mean values for each of the 21 environmental variables at the locations where the breeds were tested.

**Table S2.** Range of values for each of the 21 environmental variables at the locations where the breeds were tested.

**Table S3.** Correlations between the predicted values and the LSmeans for each breed in each region in Ethiopia.

NA= Not available or sample size less than 25.

**Figure S1.** Correlations between the predicted values and the LSmeans for each breed in each region in Ethiopia for the male growing period.


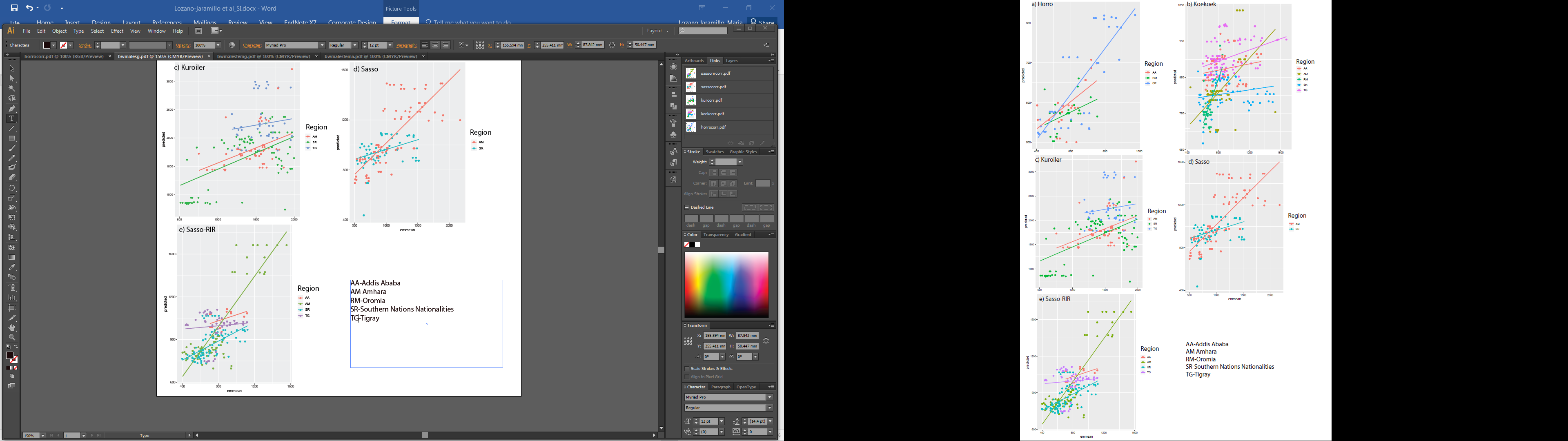


**Figure S2.** Correlations between the predicted values and the LSmeans for each breed in each region in Ethiopia for the female growing period.


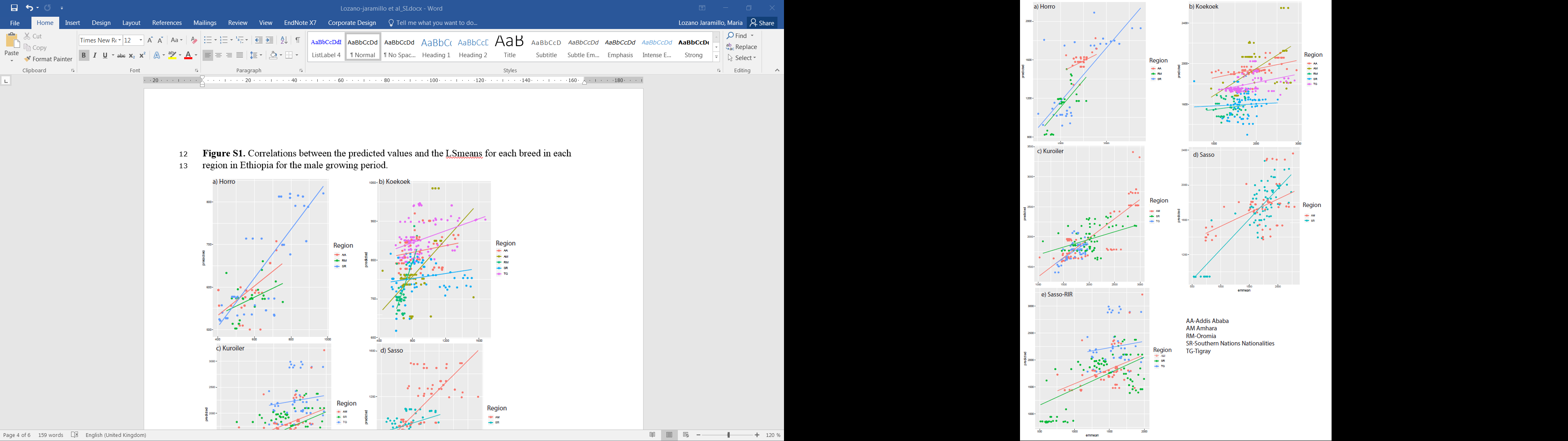


**Figure S3.** Correlations between the predicted values and the LSmeans for each breed in each region in Ethiopia for the female adult period.


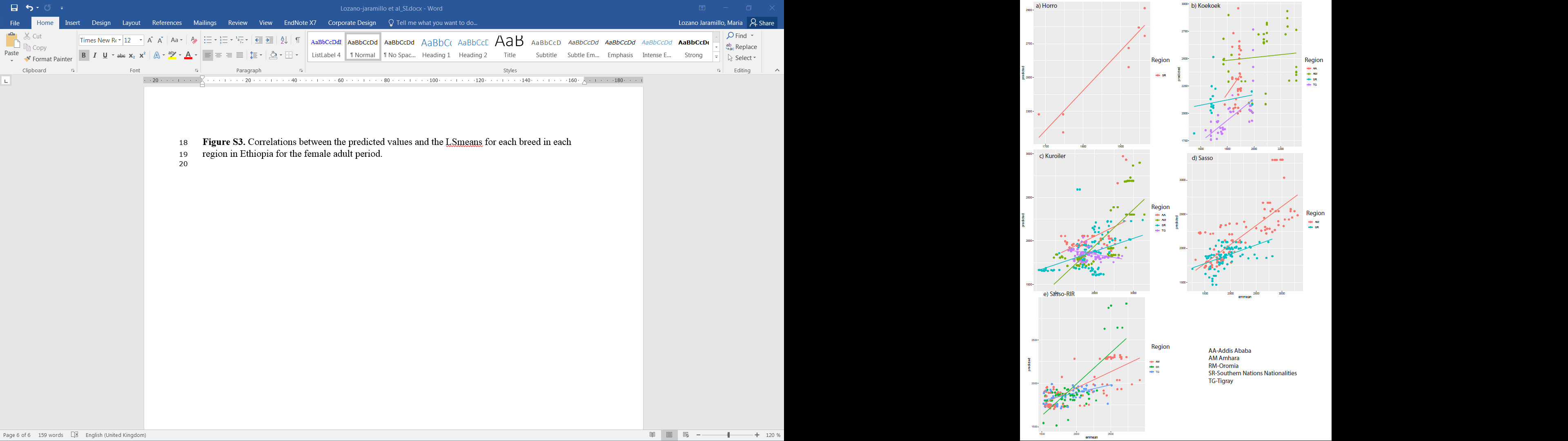

Supplement: Supplementary file 1 — Supplementary material [file 41598_2019_51910_MOESM1_ESM.docx]
